# *Citrus junos* Tanaka peel ameliorates hepatic lipid accumulation in HepG2 cells and in mice fed a high-cholesterol diet

**DOI:** 10.1186/s12906-016-1460-y

**Published:** 2016-12-03

**Authors:** Eun Ju Shin, Jae Ho Park, Mi Jeong Sung, Min-Yu Chung, Jin-Taek Hwang

**Affiliations:** 1 Korea Food Research Institute, University of Science & Technology, 1201 Anyangpangyoro, Bundang-gu, Seongnam-si, Gyeonggi-do 463-746 Republic of Korea; 2 Department of Food Biotechnology, University of Science & Technology, 217 Gajeong-ro, Useong-gu, Daejeon, 305-333 Republic of Korea

**Keywords:** Citrus junos Tanaka, Hepatic lipid accumulation, High cholesterol diet, HepG2 cell

## Abstract

**Background:**

*Citrus junos* Tanaka (yuja), a yellow-coloured citrus fruit has traditionally been consumed in Korea, Japan, and China and has been found effective in preventing certain diseases. However, the inhibitory effect of yuja on hepatic lipid accumulation has not been clearly elucidated thus far.

**Methods:**

The inhibitory effect of yuja on hepatic lipid accumulation was investigated in both cell culture and mouse models. We investigated the inhibitory effect of ethanol extract of yuja peel (YE) using HepG2 cells. We next confirmed the effect of YE in mice fed a high cholesterol diet. Animals were divided into 4 groups (*n* = 8): a normal diet group (ND), a high-cholesterol diet group (HC), high-cholesterol diet plus 1% YE (YL), high-cholesterol diet plus 5% YE (YH).

**Result:**

Seventy percent ethanolic extracts of yuja peel (YE) reduced oleic acid-induced hepatic lipid accumulation in HepG2 cells. Treatment with YE at 100, 200 μg/mL up-regulated expression levels of cholesterol metabolism-related proteins such as AMPK, ACC, PPAR-α, and CPT1 and down-regulated the expression of 3-hydroxy-3-methylglutaryl coenzyme A reductase. The hypocholesterolemic effect of YE was further confirmed in mice fed a high-cholesterol diet. Compared to ND (normal diet) mice, HC (high-cholesterol diet) mice showed increased body weight, liver fat content, liver weight, and content of total cholesterol and low-density lipoprotein (LDL) cholesterol. On the contrary, administrations of YL (HC + 1% YE) or YH (HC + 5% YE) significantly reduced body weight, liver fat content, liver weight, total cholesterol, and LDL cholesterol compared to those of only HC fed mice group. As a result of in vitro data, protein expressions of PPAR-α and CPT1 were induced in mice fed YE diet compared to HC diet but HMGCR expression was decreased.

**Conclusions:**

Yuja peel ameliorates hepatic lipid accumulation in both cell culture and mouse models and therefore, could serve as a useful supplement for hypercholesterolemia.

## Background

Lifestyle-related diseases including hepatic lipid accumulation are associated with irregular lifestyle and diet. Hepatic lipid accumulation is accompanied by excessive accumulation of cholesterol and triglycerides, thereby causing atherosclerosis, which affects the lipid‐laden blood vessels, or hepatic steatosis, or both. It is well established that low-density lipoproteins (LDLs) play a central role in the development of atherosclerosis. LDL promotes formation of fatty streaks, a key event in early atherosclerosis and induces the uptake of oxidized LDL by macrophages and smooth muscle cells. A negative correlation has been demonstrated between LDL and high-density lipoproteins (HDLs). High level of HDL can inhibit LDL oxidation by various mechanisms [1]. Several studies have shown that natural ingredients are effective in preventing hypercholesterolemia by suppressing LDL oxidation [2]. For example, cocoa polyphenols inhibit LDL oxidation, thereby suppressing the formation of atherosclerosis. The intake of dairy cocoa powder was shown to reduce LDL oxidation in humans [2]. It was also reported that vitamin E supplementation inhibits LDL accumulation in arterial diseases. A clinical study reported that almonds significantly reduced total cholesterol (range 8-12%) and LDL cholesterol (range 9–15%) [3]. Therefore, reduction of total cholesterol and LDL cholesterol levels may be a significant strategy for preventing atherosclerosis.

Another mechanism of preventing hypercholesterolemia includes targeting enzymes including AMPK and 3-hydroxy-3-methylglutaryl coenzyme A reductase (HMGCR). AMPK is a metabolic protein, which plays a central role in lipid metabolism and inhibits anabolic pathways, including cholesterol synthesis by targeting HMGCR [4]. HMGCR is a rate-limiting enzyme in cholesterol synthesis pathway; it is suppressed by cholesterol derived from the degradation of LDL via the LDL receptor [5]. HMGCR is inhibited by statins, which upregulate the expression of LDL receptors in the liver, resulting in increased catabolism of plasma LDL and decreased plasma cholesterol [5]. Thus, AMPK and HMGCR enzymes are the targets of various cholesterol-lowering natural ingredients.

*Citrus junos* Tanaka, also known as yuja is a yellow-coloured citrus fruit that has been reported to exhibit beneficial health effects against oxidative stress and inflammation [6, 7]. In our previously paper, several active compounds found in yuja exist as rutin, quercetin, tangeretin, naringin and hesperidin [6]. In addition, our studies have also reported the beneficial health effects of yuja, such as potent antidiabetic, anticancer, and anti-inflammatory effects, both in in vitro and in vivo [6, 7]. The present study investigated the effects of yuja on hepatic lipid accumulation. A 70% ethanolic extract of yuja peel was examined for its effects on oleic acid-induced hepatic lipid accumulation, AMPK activation, and HMGCR expression in HepG2 cells. Further, the same ethanolic extract was evaluated in mice fed a high-cholesterol diet, because mice fed high-cholesterol diet is widely used to study hepatic lipid metabolism.

## Methods

### Reagents

MTT (3-(4,5-dimethylthiazol-2-yl)-2,5-diphenyltetrazolium bromide) and Oil Red O were purchased from Sigma-Aldrich (St. Louis, MO, USA). Triglyceride (TG), Total Cholesterol (TC), Glutamic Oxaloacetic Transaminase (GOT), Glutamic Pyruvic Transaminase (GPT), and Alkaline Phosphatase (ALP) kits were purchased from Asan Pharmaceutical company (Seoul, Republic of Korea). Fatty Acid Synthase (FAS), Carnitine Palmitoyl Transferase-1 (CPT-1), and Peroxisome Proliferator-Activated Receptor-α (PPAR-α) antibodies were purchased from santacruz Biotechnology Inc. (Santa Cruz, CA, USA), 3-Hydroxy-3-Methylglutaryl-Coenzyme A Reductase (HMGCR) was purchased from Cell Signaling (Beverly, MA, USA). β-Actin was purchased from Bethyl Laboratories (Montgomery, TX, USA).

### Preparation of Yuja extracts

Yuja was provided by Goheung Country Office (Goheung, Republic of Korea), where a voucher specimen was deposited. Yuja peel extracts (YE) were prepared as described previously [7]. Briefly, yuja peels were dissolved in a 10-fold volume of 70% ethanol by shaking for 24 h at 25 °C, and precipitates were removed by centrifugation at 8000xg for 30 min. Supernatants were dried using freeze dryer. Yuja extract was dissolved in DMSO and used to treat HepG2 cells and mice fed high fat diet. The cells were incubated with 1% BSA-supplemented low-glucose DMEM (None), 0.5 mM oleic acid (OA) in 1% BSA-supplemented low-glucose DMEM, and 0.5 mM OA in DMEM with 50, 100, and 200 μg/mL YE as a treatment group for 24 h.

### Cell culture and sample treatment

HepG2 cells were purchased from the American Type Culture Collection (Mannassas, VA, USA). These cells were grown in high-glucose Dulbecco’s modified Eagle’s medium (DMEM) supplemented with 10% fetal bovine serum (FBS) and antibiotics, which were purchased from Welgene Inc. (Daegu, Republic of Korea). The cells were maintained at 37 °C in a humidified atmosphere under 5% CO_2_. When processing the sample on the cells, cells were treated with 1% BSA in low-glucose DMEM (None), 0.5 mM oleic acid (OA) in 1% BSA-supplemented low-glucose DMEM, and 0.5 mM OA in DMEM with 50, 100, and 200 μg/mL YE as a treatment group for 24 h.

### Cytotoxicity (MTT assay)

HepG2 cells were cultured in 24-well plates and treated with YE at the indicated concentrations. Then, 10 μL of MTT solution (5 mg/mL in PBS) was added and incubated for 3 h. After removing the medium, the cells were dissolved in dimethylsulfoxide (DMSO), and 100 μL of supernatant was transferred to 96-well plates. Absorbance was measured at 540 nm using microplate reader (Molecular Device Co., Sunnyvale, CA, USA).

### Oil Red O staining

HepG2 cells were cultured in 24-well plates and then treated with YE for 24 h. After treatment, they were stained with Oil Red O to measure lipid droplet accumulation, washed with 200 μL of PBS, and fixed with 200 μL of 4% formaldehyde for 15 min, at room temperature (RT). The cells were then washed three times with PBS, incubated with 200 μL of 60% isopropanol for 5 min, and then stained with 200 μL of 0.1% Oil Red O staining solution for 60 min at RT. The cells were further washed 3 times with 1 mL of water. The images of these cells were captured by microscopy (Olympus, Tokyo, Japan). To measure lipid accumulation, the cells were dissolved in isopropanol for 10 min. The dissolved dye was transferred to 96-well plates and the absorbance was measured at 510 nm.

### Western blot assay

The proteins were harvested in RIPA buffer (Elpis, Daejeon, Republic of Korea) containing a protease inhibitor and a phosphatase inhibitor (Roche, Basel, Switzerland). After this treatment, the protein was quantified using Bicinchoninic Acid (BCA) methods. The protein samples (20 μg) were loaded onto 10% Bis-Tris gel and transferred to nitrocellulose membranes. The membrane was blocked using 5% skim milk solution for 1 h and probed with each antibody at 4 °C overnight. After triplicate washing with PBS, the membrane was incubated with horseradish peroxidase-conjugated secondary antibodies for 1 h. It was washed again with PBS and the protein expression was detected by chemiluminescence methods.

### Animal fed a high-cholesterol diet

Male C57BL/6 J mice (3 weeks old) were housed at the Korea Food Research Institute (KFRI) in a climate-controlled environment (24 °C at 50% relative humidity) with a 12 h light/dark cycle. After 1 week of adaptation, the mice were randomly divided into four groups (*n* = 8): N (Normal diet), HC (high-cholesterol diet), YL (HC + 1% YE), and YH (HC + 5% YE). D12336 diet (high-cholesterol diet, Purified diet to match Paigen’s atherogenic rodent diet, Research Diets, Inc.) was used as HC. The composition of the high cholesterol diet was as follow: 46% carbohydrate, 16% fat, 21% protein, 12.5% cholesterol, 0.5% cholic acid, mineral mixture and vitamin mixture (source of carbohydrate = maltodextrin, sucrose and corn starch; source of fat = soybean oil, cocoa butter and coconut oil; source of protein = casein, soy protein and DL-methionine, source of mineral mixture = calcium carbonate, sodium chloride, potassium citrate; vitamin mixture = choline bitartrate). The mice were fed these diets for 10 weeks with free access to autoclaved tap water in cages. The total daily intake and weight of mice were recorded every week for up to 10 weeks. At the end of the experiment, animals were sacrificed to collect their blood and liver tissue samples, which were then stored at −70 °C. Our experimental protocol was approved by the Institutional Animal Care and Use Committee of the Korea Food Research Institute.

### Hematoxylin and eosin (H&E) staining

Liver tissues were fixed using 4% formaldehyde solution and cut into 4-μm-thick portions, which were stained with hematoxylin and eosin, and images were captured using a microscope (Olympus, Tokyo, Japan).

### Measurement of enzyme and lipid change in blood

The collected blood samples were centrifuged at 12000 rpm for 15 min at 4 °C. After centrifugation, the serum was transferred to a new centrifuge tube and then stored at −70 °C. Blood serum levels of TG, TC, HDL-cholesterol, GOT, GPT, and ALT were analysed according to the manufacturer’s protocol.

### Statistical analysis

Results obtained from at least three independent in vitro studies were expressed as mean ± standard deviation (SD). The results were determined by nonparametric methods using the SPSS computer-based statistics programs (Ver. 20; SPSS Inc., Chicago, IL, USA). In case of in vivo studies using mice, the results are expressed as mean ± standard error of mean (SE). Statistical differences between mean values were evaluated by one-way analysis of variance (ANOVA) followed by Bonferroni test. A *P* value of <0.05 was considered statistically significant.

## Results

### Inhibition of OA-induced hepatic lipid accumulation by YE without toxicity in HepG2 cells

**Fig. 1:**
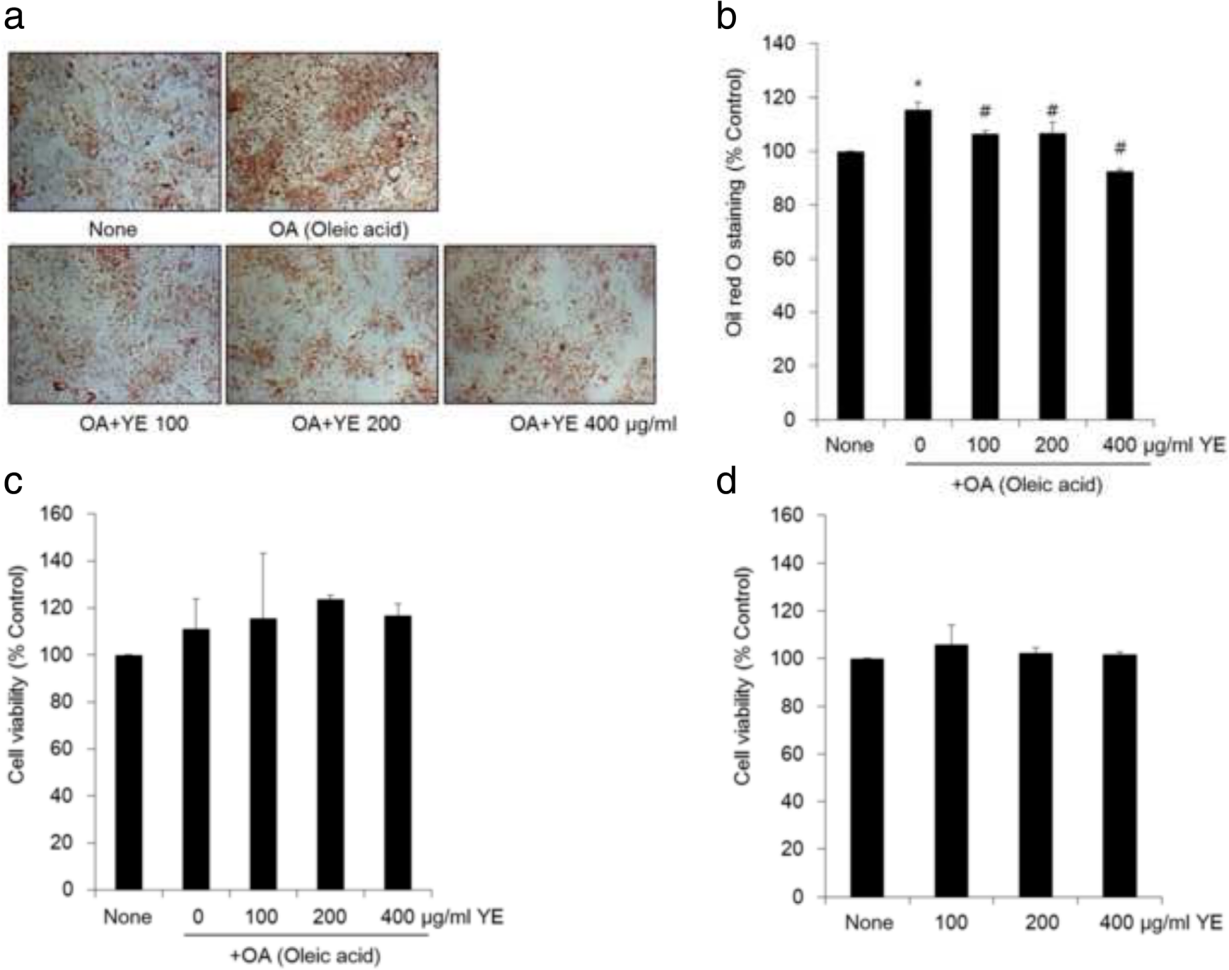
Effects of 70% ethanolic extracts of yuja peel (YE) on hepatic lipid accumulation in and viability of HepG2 cells. HepG2cells were treated for 24 h with YE in the presence or absence of oleic acid (OA), and then lipid accumulation was measured by Oil-Red O staining (**a**, **b**). Cytotoxicity was measured by the MTT assay (**c**, **d**). All results are expressed as the mean ± SD. ^*^ *p* < 0.05 vs. control (None); ^#^ *p* < 0.05 vs. OA

To determine the inhibitory effect of YE on hepatic lipid accumulation, lipid accumulation was evaluated using Oil red O staining of HepG2 cells. As shown in Fig. 1a and b, treatment with 0.5 mM OA significantly induced lipid accumulation by about 16% compared to control (untreated HepG2 cells). Co-treatment with 0.5 mM OA and YE (100, 200 and 400 μg/ml) significantly inhibited up to 23% OA-induced lipid accumulation in a dose-dependent manner. Under similar conditions, the effect of YE on cell viability was also determined by MTT assay. There were no significant differences in cell viability, indicating that the tested concentrations of YE did not affect cell viability (Fig. 1c). Similar results were obtained for treatment with only YE (Fig. 1d). These results suggest that YE has preventive effects against OA-induced hepatic lipid accumulation without cell toxicity.

### Prevention of hepatic lipid accumulation by YE via modulation of AMPK signalling HMGCR, PPAR-α, and CPT-1 in HepG2 cells

**Fig. 2:**
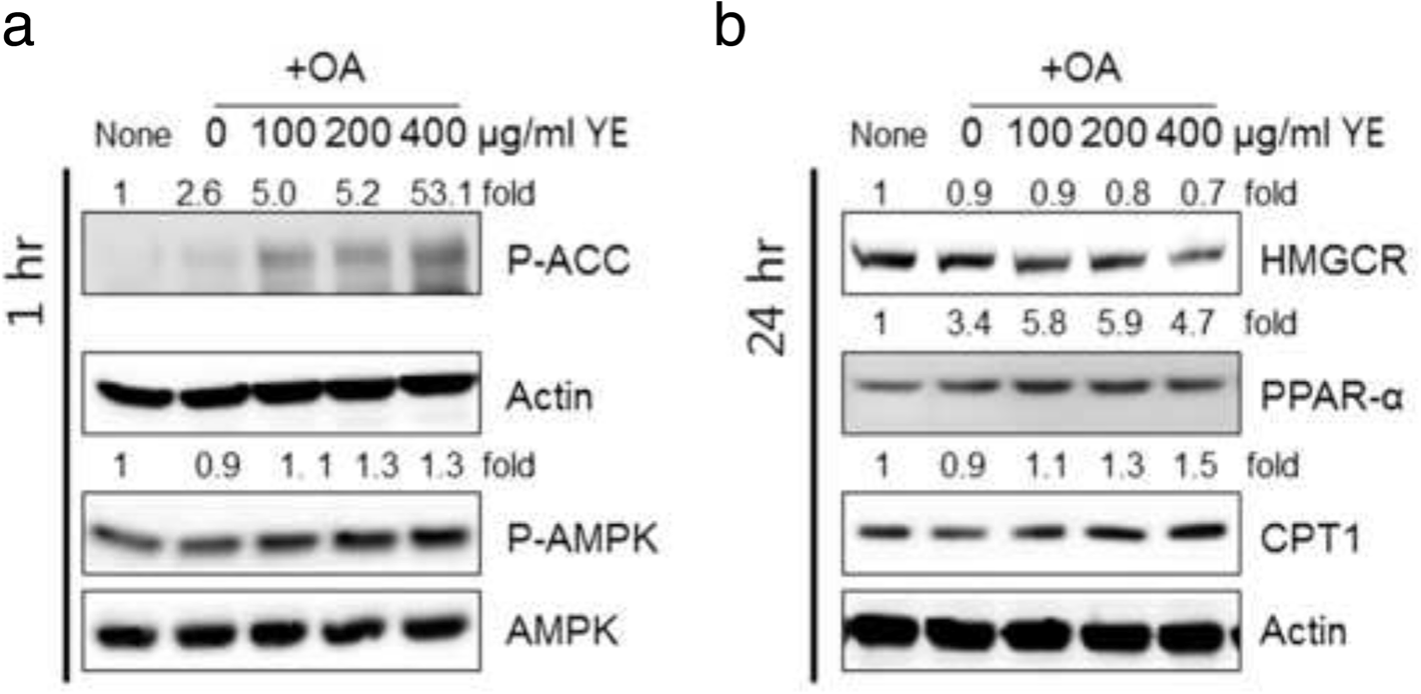
Effects of 70% ethanolic extracts of yuja peel (YE) on AMPK signalling and expression of proteins associated with cholesterol metabolism. HepG2cells were treated for 1 h with YE in the presence or absence of oleic acid, and then western blot analysis was performed with phospho-specific ACC, AMPK, normal AMPK, and beta-actin antibodies (**a**). HepG2cells were treated for 24 h with YE in the presence or absence of oleic acid, followed by western blot analysis with 3-hydroxy-3-methylglutaryl coenzyme A reductase (HMGCR), peroxisome proliferator-activated receptor (PPAR)-α, carnitine palmitoyltransferase (CPT)-1, and beta-actin antibodies (**b**)

AMPK is a potential modulator of metabolism of lipid accumulations via up-regulation of fatty acid oxidation and down-regulation of lipogenesis, glucose production, and protein synthesis [4]. In view of this information, the effect of YE on AMPK signalling was investigated. HepG2 cells were incubated with OA, as well with OA and YE for 1 h. As shown in Fig. 2a, YE stimulated the phosphorylation (Thr172) of AMPK and its major downstream targeting enzyme, ACC. These results indicated that the AMPK signalling pathway is the potential target of YE, thereby preventing hepatic lipid accumulation in HepG2 cells. To investigate the specific downstream of AMPK activation, the effects of YE on the expression of lipid metabolism-related proteins were evaluated, such as HMGCR, PPAR-α, and CPT1, which are known for fatty acid β-oxidation [8, 9]. Treatment with OA alone did not affect HMGCR, PPAR-α, and CPT1 expression. However, co-treatment with OA and YE reduced HMGCR expression. In contrast, co-treatment with OA and YE induced PPAR-α and CPT1 expression (Fig. 2b). These results indicated that YE inhibited OA-induced hepatic lipid accumulation via AMPK signalling, in particular, by stimulating PPAR-α and CPT1, β-oxidation-related proteins and reducing HMGCR proteins involved in lipogenesis.

### Inhibition of increases in body weight and liver weight by YE in HC-fed mice

**Fig. 3:**
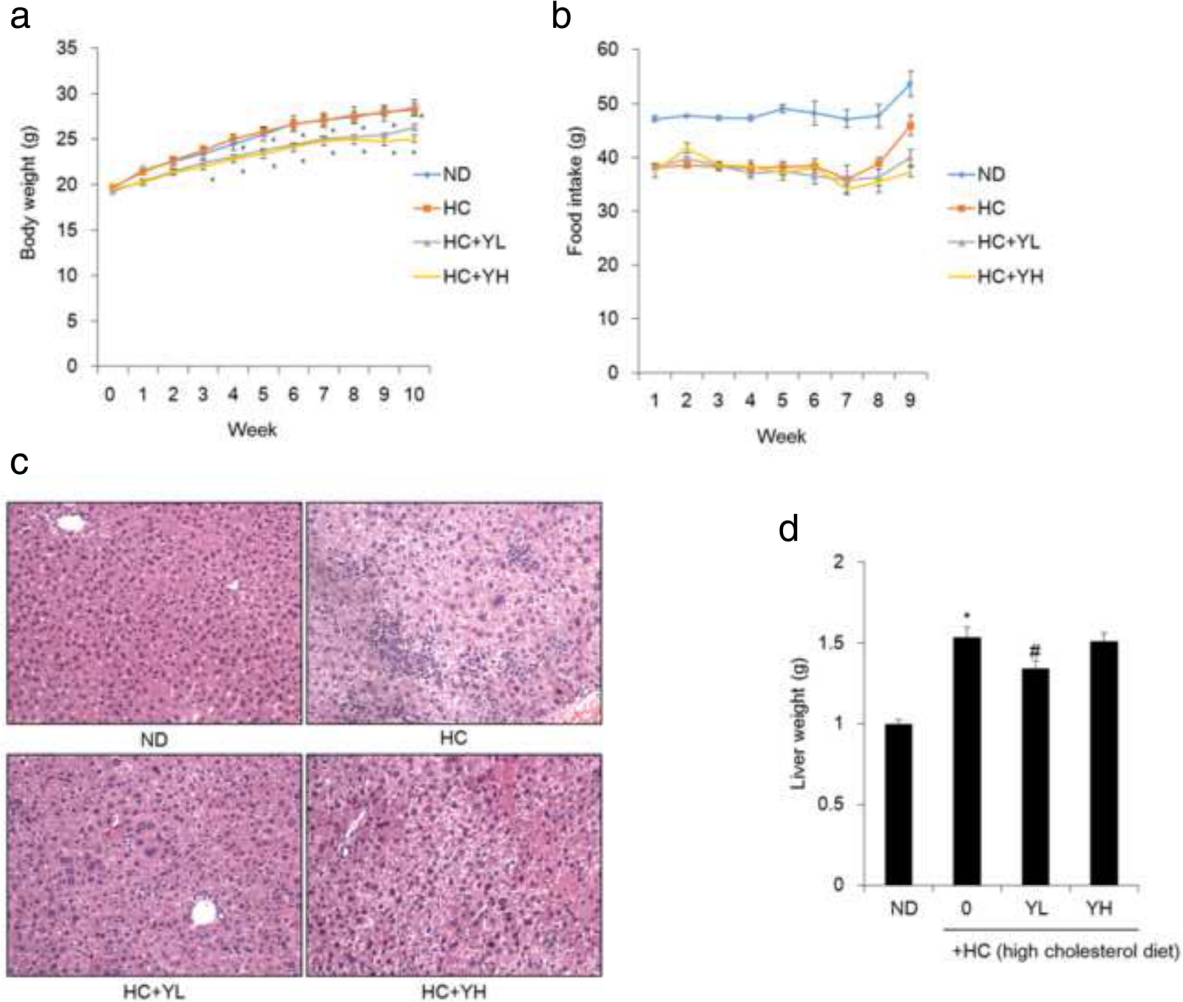
Effects of 70% ethanolic extracts of yuja peel (YE) on body weight and liver weight in high-cholesterol diet (HC)-fed mice. Experimental mice were fed normal diet, HC, and HC supplemented with 1% (YL) or 5% (YH) YE for 10 weeks. Body weight (**a**), food intake (**b**), liver fat accumulation (**c**), and liver weight (**d**) were measured, as described in materials and methods. Results are expressed as mean ± SE. ^*^ *p < 0.05* vs. *HC*

To further examine the hypocholesterolaemic effects of YE, studies were carried out using HC-fed mice. High cholesterol diet caused fatty liver in this mice model. While the food intake in normal group is higher than in HC group (Fig. 3b), As Fig. 3a, the body weight between the normal and HC groups did not differ significantly (28.4 ± 0.9 vs. 28.3 ± 0.7 g, normal vs. HC group). YL (HC + 1% YE) and YH (HC + 5% YE) group mice had significantly lower body weights than normal or HC group mice (26.2 ± 0.3, 25.1 ± 0.4, respectively, vs. 28.3 ± 0.7 g, YL, YH vs. HC group). In addition, YL and YH group showed inhibited fat droplet accumulation compared with HC group in the liver (Fig. 3c). And YL group mice showed reduced liver weight compared to HC group mice (Fig. 3d). These result suggested that administration of YE protected the mice from HC-induced fatty liver.

### Attenuation of serum biomarkers increased HC by YE in mice

**Fig. 4:**
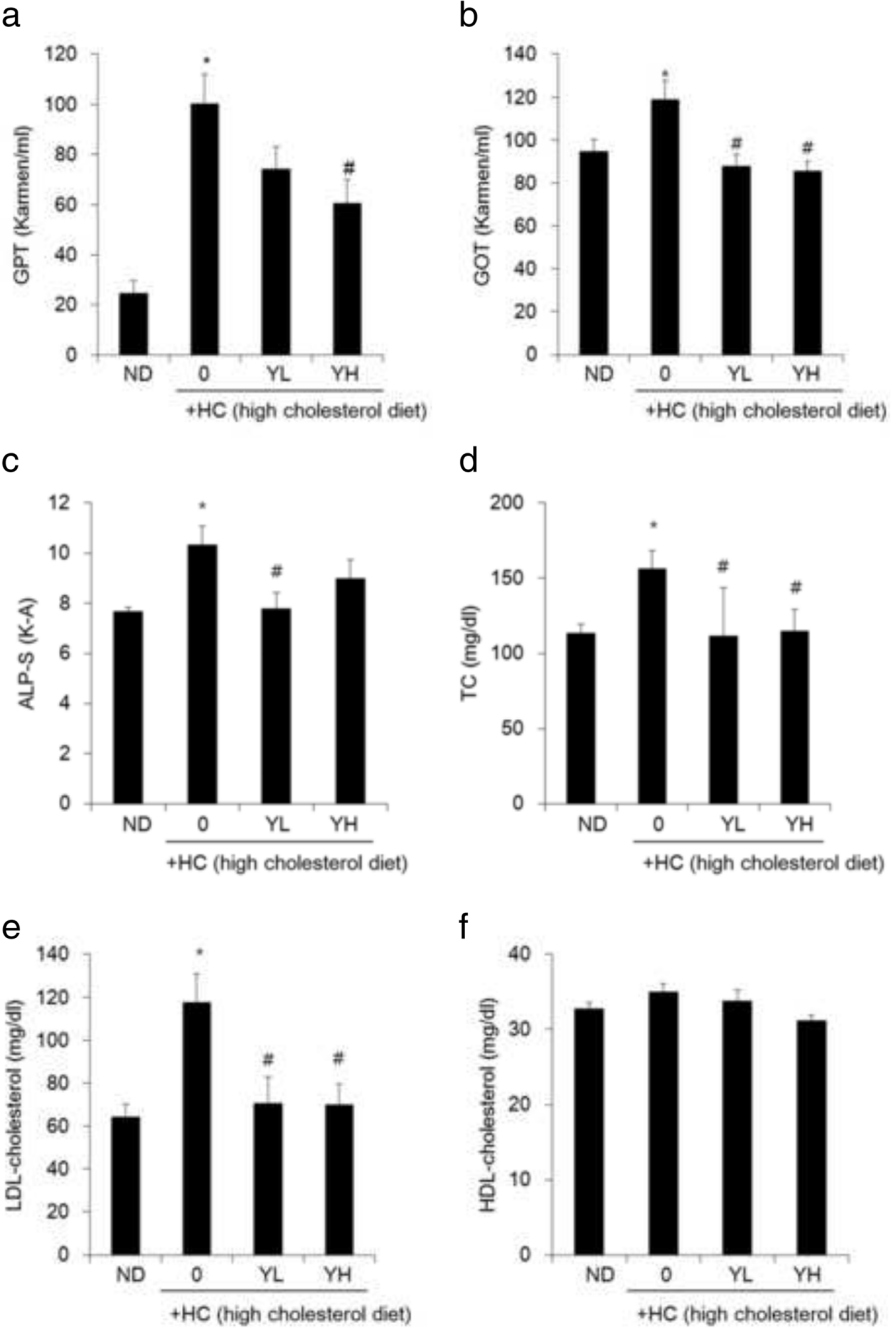
Effects of 70% ethanolic extracts of yuja peel (YE) on biochemical parameters related to cholesterol metabolism. Experimental mice were fed normal diet, high-cholesterol-diet (HC), and HC supplemented with 1% (YL) or 5% (YH) YE for 10 weeks. Serum levels of (**a**); glutamic pyruvic transaminase (GPT), (**b**); glutamic oxaloacetic transaminase (GOT), (**c**); alanine phosphatase (ALP-S), (**d**); total cholesterol (TC), (**e**); low-density lipoprotein (LDL), (**f**); and high-density lipoprotein (HDL) were measured, as described in materials and methods. Results are expressed as mean ± SE (*n* = 8). ^*^ *p* < 0.05 vs. ND; ^*#*^ *p* < 0.05 vs. HC

To examine the effects of YE on serum biomarker, the serum levels of GPT, GOT, and ALP were evaluated. The levels of GPT, GOT, and ALT, which are indicative of liver activity, increased in the HC group. In contrast, the levels of GPT, GOT and ALT significantly decreased in the YL and YH groups (Fig. 4a, b, c). Next, we analysed serum cholesterol, HDL, and LDL levels. Along with hepatic lipid accumulation, cholesterol and LDL levels of HC group dramatically enhanced compared with normal diet group. Supplementation with YL and YH attenuated the HC-induced blood levels of TC and LDL, with little effect on HDL cholesterol levels (Fig. 4d, e, f). These results indicated that YE exerts hypocholesterolaemic effects against HC-induced lipid accumulation by improving the blood lipid levels and liver activity.

### Inhibition of FAS, HMGCR and induction of PPAR-α hepatic protein expressions by YE in mice

**Fig. 5:**
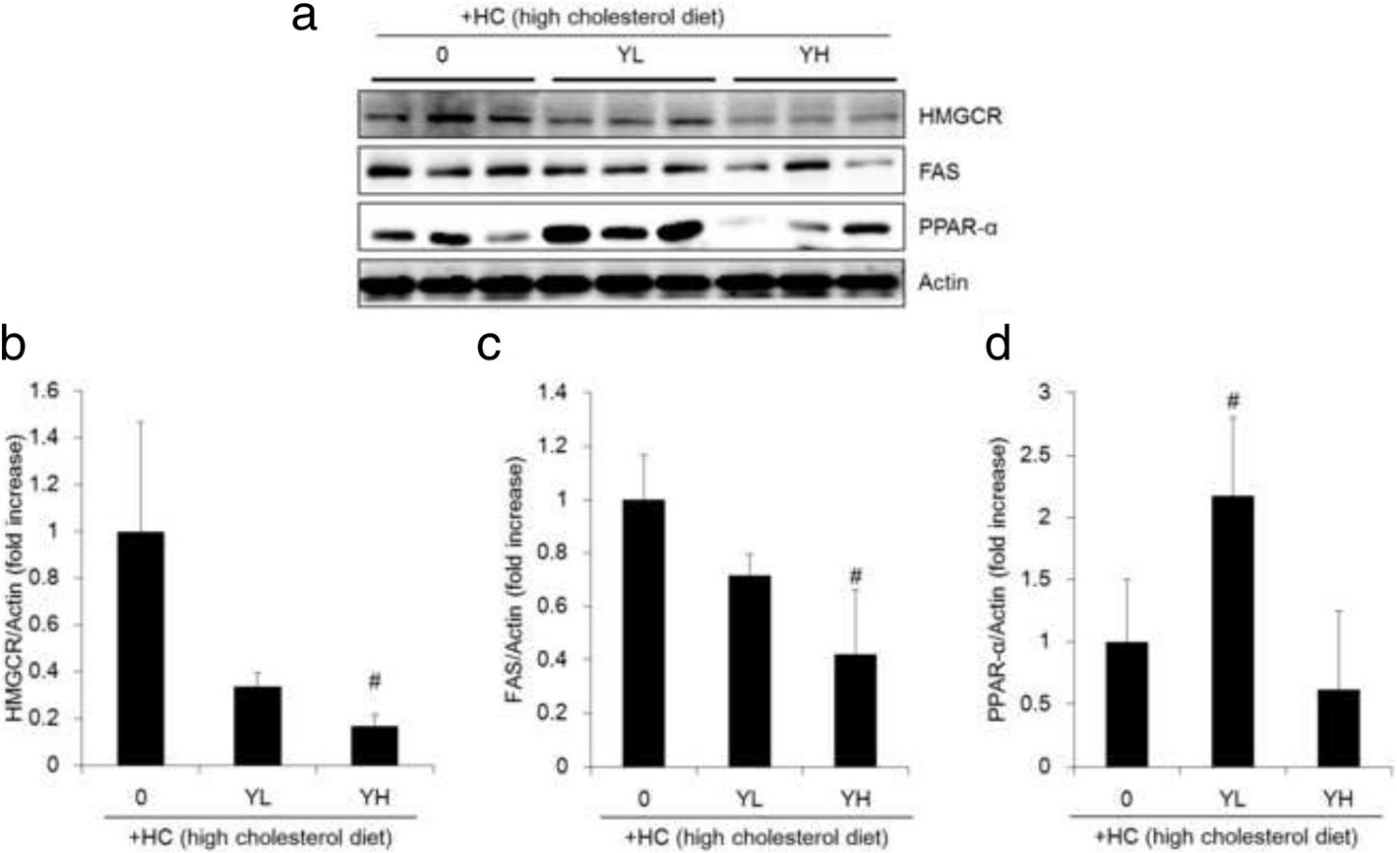
Effects of 70% ethanolic extracts of yuja peel (YE) on protein expressions associated with cholesterol metabolism in liver tissue. Experimental mice were fed normal diet, high-cholesterol-diet (HC), and HC supplemented with 1% (YL) or 5% (YH) YE for 10 weeks. Three livers were randomly chosen in each group. Hepatic protein expressions of HMGCR, FAS, PPARα and actin were determined by immunoblotting (**a**). And relative densities of each bands were quantified (**b**, **c**, **d**). Results are expressed as mean ± SD. ^*#*^ *p* < 0.05 vs. HC

We further examined protein expressions related to lipid metabolism in liver tissue. Consistent with the vitro protein results in HepG2 cells, administration of HC with YL and YH groups inhibited HMGCR and FAS protein expressions compared to HC group (Fig. 5a, b, c). On the other hands, only administration of YL increased expressions of PPARα which are involved in β-oxidation (Fig. 5a, d), but not in YH groups.

Taken together, both in vitro and in vivo data showed that YE attenuated hepatic lipid accumulation by multiple mechanisms such as fatty acid oxidation, lipogenesis.

## Discussion

In the present study, the hypocholesterolemic effect of YE was evaluated in a cell culture system and in mice fed an HC. It was also demonstrated that YE reduces hepatic lipid accumulation, HMGCR expression, and induces the phosphorylation of AMPK without cytotoxicity in HepG2 cells. Furthermore, 1% YE (YL) or 5% YE (YH) reduces the body weight, liver fat content, liver weight, total cholesterol, and LDL cholesterol in mice fed an HC.

HepG2 cells have been proposed to be a valuable model for screening of hypocholesterolemic agents [10]. To determine the safety of plant extracts, a cytotoxicity assay was performed before evaluating physiological activities in vivo studies. Therefore, the cytotoxicity of YE in HepG2 cells was evaluated using the MTT assay. No significant cytotoxicity was observed (Fig. 1). Treatment with YE was also shown to reduce OA-stimulated hepatic lipid accumulation in HepG2 cells. In addition, treatment with YE induces protein expressions of AMPK, PPAR-α, CPT1 and inhibits HMGCR expression in OA-mediated HepG2 cells. Lovastatin and simvastin have been used as inhibitors of HMGCR, a major target for cholesterol synthesis [11]. HMGCR catalyses the conversion of HMG-CoA to mevalonic acid, which is a rate-limiting step in cholesterol biosynthesis [5, 11]. Therefore, YE may inhibit the conversion of HMG-CoA to mevalonic acid by HMGCR without any cytotoxicity. In addition, AMPK was phosphorylated by YE in HepG2 cells. AMPK is an upstream regulator of HMGCR and many investigators have focused on the role of AMPK for the development hypocholesterolemic compounds [4]. For example, several plant extracts and ingredients have been reported to be effective in inhibiting HMGCR and AMPK, such as caffeine, berberine, and *Artemisia scoparia* extract [12–14]. This is in agreement with our result that the hypocholesterolemic effect of YE is modulated via the AMPK/HMGCR pathways.

Based on the above results from HepG2 cell culture, an animal study was conducted using mice fed an HC. 1% YE (YL) or 5% YE (YH) ameliorates hypercholesterolemia by decreasing weight gain, liver fat content, liver weight, total cholesterol, and LDL cholesterol content. Liver weight and ALT values of YL groups was more effective than that of YH group. On the other hand, the overall results of YH groups were better than that of YL group. Several recent studies demonstrated that consumption of plant extracts decreases the level of LDL, an important factor for cardiovascular diseases. Increased level of cholesterol results in atherosclerosis due to the increased lipid peroxidation [14–16]. Thus, LDL peroxidation is an important target for the prevention of atherosclerosis and can be prevented by antioxidants. Owing to minimal side effects, natural products are reported as an alternative for the prevention of atherosclerosis. Several plants have antioxidative properties. In previous studies, *Biebersteinia multifida* DC (Geraniaceae) was shown to have an antioxidant effect and significantly decreased the level of LDL [17]. The antioxidant ability of this plant possibly facilitates its hypocholesterolemic effect. Our previous study has shown that yuja peel has antidiabetic activity and has several phenolic compounds, including rutin, quercetin, tangeretin, naringin, and hesperidin [6, 7]. Most of these compounds have excellent anti-oxidant abilities. Citrus flavonoids have been reported to have the hypocholesterolemic effects [18]. Our previous study showed that tangeretin could reduce the levels of circulating lipid mediators, including total cholesterol, in obese mice [19]. In the present study, YE reduced weight gain, liver fat content, liver weight, total cholesterol, and LDL cholesterol content in mice fed an HC. Besides, YE also inhibited HMGCR, FAS and stimulated PPARα expressions in liver tissue compared to HC-fed groups like in vitro results. These results indicate that YE have the potential to improve hypercholesterolemia, which could be attributed to the antioxidant effect of polyphenol compounds. These compounds have an aromatic-ring structure with various substitutions [20]. The 3-hydroxyl group on the aromatic ring is a hydrogen donor and has an antioxidant effect [20]. Therefore, it is speculated that the 3-hydroxyl group of flavonoids that are abundant in YE is important for the hypocholesterolemic effect.

## Conclusions

In this study, we demonstrate the hypocholesterolemic effect of 70% ethanolic extract of yuja peel in HepG2 cells. A 70% ethanolic extract of yuja peel also reduced weight gain, liver fat content, liver weight, total cholesterol, and LDL cholesterol content. Therefore *Citrus junos* Tanaka (yuja) may be useful for preventing atherosclerosis.
